# Response of net primary production to land use and climate changes in the middle‐reaches of the Heihe River Basin

**DOI:** 10.1002/ece3.5068

**Published:** 2019-03-30

**Authors:** Xingyuan Xiao, Xiubin Li, Tao Jiang, Minghong Tan, Minyue Hu, Yaqun Liu, Wen Zeng

**Affiliations:** ^1^ College of Geomatics Shandong University of Science and Technology Qingdao China; ^2^ Key Laboratory of Land Surface Pattern and Simulation, Institute of Geographic Sciences and Natural Resources Research Chinese Academy of Sciences Beijing China; ^3^ University of Chinese Academy of Sciences Beijing China

**Keywords:** anthropogenic‐natural coupled effects, carbon sequestration, ecosystem sustainability, remote sensing, spatial differential response, vegetation primary productivity

## Abstract

Net primary production (NPP) supplies matter, energy, and services to facilitate the sustainable development of human society and ecosystem. The response mechanism of NPP to land use and climate changes is essential for food security and biodiversity conservation but lacks a comprehensive understanding, especially in arid and semi‐arid regions. To this end, taking the middle‐reaches of the Heihe River Basin (MHRB) as an example, we uncovered the NPP responses to land use and climate changes by integrating multisource data (e.g., MOD17A3 NPP, land use, temperature, and precipitation) and multiple methods. The results showed that (a) land use intensity (LUI) increased, and climate warming and wetting promoted NPP. From 2000 to 2014, the LUI, temperature, and precipitation of MHRB increased by 1.46, 0.58°C, and 15.76 mm, respectively, resulting in an increase of 14.62 gC/m^2^ in annual average NPP. (b) The conversion of low‐yield cropland to forest and grassland increased NPP. Although the widespread conversion of unused land and grassland to cropland boosted both LUI and NPP, it was not conducive to ecosystem sustainability and stability due to huge water consumption and human‐appropriated NPP. Urban sprawl occupied cropland, forest, and grassland and reduced NPP. (c) Increase in temperature and precipitation generally improved NPP. The temperature decreasing <1.2°C also promoted the NPP of hardy vegetation due to the simultaneous precipitation increase. However, warming‐induced water stress compromised the NPP in arid sparse grassland and deserts. Cropland had greater NPP and NPP increase than natural vegetation due to the irrigation, fertilizers, and other artificial inputs it received. The decrease in both temperature and precipitation generally reduced NPP, but the NPP in the well‐protection or less‐disturbance areas still increased slightly.

## 1 INTRODUCTION

Net primary production (NPP) is the net carbon sequestration of terrestrial plants, namely the difference between photosynthesis production and respiration consumption (Matsushita & Tamura, 2002; Milesi, Elvidge, Nemani, & Running, 2003; Potter, Klooster, & Genovese, 2012; Running et al., 2004). NPP supplies food, fuel, and fiber for the growing human population (Potter et al., 2012; Running et al., 2004), and provides energy and substances for most biological processes in terrestrial and aquatic ecosystems (Chitale, Tripathi, Behera, Behera, & Tuli, 2012; Lieth & Whittaker, 1975), which is fundamental to human survival and development, food security, and biodiversity conservation. Furthermore, NPP supports the regulation and cultural services of ecosystem, for example, climate regulation, air purification, soil erosion control, aesthetic value, leisure, and ecotourism (Liu, Song, & Mu, 2017; MA, 2005; Milesi et al., 2003). Thus, NPP is an important part of carbon cycle, carbon budget, and global changes, and an effective indicator for ecosystem sustainability (Cramer & Field, 1999; Field, Behrenfeld, Randerson, & Falkowski, 1998).

Ecosystem NPP is jointly affected by human activities and environmental factors (Gao, Li, & Yang, 2003; MA, 2005; Melillo et al., 1993; Zhao & Running, 2010). With population and economic growth, human‐induced land use change has become one of the most important anthropogenic factors affecting NPP (Gao et al., 2003; Han, Song, & Deng, 2016; Peng et al., 2017; Song & Deng, 2017). To meet the increasing human demand for materials and energy, large‐scale land cover has been artificially altered worldwide (Liu, Song, & Deng, 2019). For example, urban land expansion caused the reduction of cropland, forest, and grassland (Jiang, Xin, Li, & Tan, 2016; Liu et al., 2019; Tan, 2014; Tan, Li, Xie, & Lu, 2005), and cropland reclamation led to deforestation and grassland reduction (Song & Deng, 2017; Song & Zhang, 2015), both of which have decreased the NPP in natural ecosystems. However, most of the NPP produced by agro‐ecosystem was consumed by humans and had limited contribution to maintaining ecosystem sustainability (Erb, Krausmann, Lucht, & Haberl, 2009; Haberl et al., 2007; Imhoff et al., 2004). In contrast, returning or abandoning cropland to forest and grassland, forest transitions, and migration‐derived vegetation restoration has promoted ecosystem NPP (Li & Li, 2017; Li, Sun, Tan, & Li, 2016; Li, Li, Tan, & Wang, 2017; Song & Zhang, 2015; Wang et al., 2016).

Climate change characterized by global warming and increased spatiotemporal differences in precipitation is the main environmental factor affecting NPP (Chitale et al., 2012; Fu, Randerson, & Moore, 2016; MA, 2005; Melillo et al., 1993; Munir, Perkins, Kaing, & Strack, 2015; Senior, Hill, Pliego, Goode, & Edwards, 2017). Global warming can nonlinearly affect vegetation photosynthesis and respiration, leading to a spatiotemporal heterogeneity in NPP variation (Ganjurjav et al., 2018; Gao et al., 2014; Leon‐Sanchez, Nicolas, Nortes, Maestre, & Querejeta, 2016; Munir et al., 2015). As water is the key to NPP accumulation, spatiotemporal changes in precipitation patterns will inevitably alter the quantity and pattern of ecosystem NPP (Lang, Song, & Zhang, 2017; Reeves, Moreno, Bagne, & Running, 2014). NPP variations reflect the interactions (e.g., impacts and responses) between land use and climate changes, and ecosystems (Piao et al., 2012; Reeves et al., 2014). Thus, scholars and policy makers should comprehensively understand the response mechanisms of NPP to land use and climate changes if we are to achieve food security and ecosystem sustainability.

Net primary production is mainly estimated by ground observations and model simulations. Field surveys and continuous observations at fixed points or FLUXNET can accurately assess local NPP and environmental factors, but it is difficult to reflect large‐scale spatial characteristics. Therefore, various models are generally used to simulate NPP at the macroscale (Cramer & Field, 1999; Cramer et al., 1999; Kicklighter et al., 1999). Climate statistics models (e.g., Miami, Thornthwaite Memorial, and Chikugo models) estimate NPP distribution based on the empirical regression relationships between NPP and climate factors (Gang et al., 2015; Lieth, 1972; Uchijima & Seino, 1985). Although these models are easy‐to‐use, they are less accurate as empirical relationships vary with time and space. Biological process models (e.g., CENTURY, TEM, BEPS, InTEC, CARAIB, DLEM, and ORCHIDEE models) estimate NPP based on the processes and mechanisms of biomass accumulation (Liu, Chen, Cihlar, & Park, 1997; McGuire et al., 1997; Parton et al., 1993; Piao et al., 2012; Tian et al., 2010; Warnant, Francois, Strivay, & Gerard, 1994). Different process parameters used in these models cause less comparability in simulation results. Moreover, the difficulty of obtaining spatialized parameters increases the uncertainty of model performance on large scales. Since the convenient data accessibility, light use efficiency models (e.g., C‐Fix, CASA, GLO‐PEM, SDBM, and TURC models) based on remote sensing have been widely used for large‐scale and long‐term NPP estimation (Bonan, 1995; Nayak, Patel, & Dadhwal, 2010; Potter et al., 1993; Ruimy, Saugier, & Dedieu, 1994; Zhu, Pan, He, Yu, & Hu, 2006). The MOD17 NPP product of Moderate Resolution Imaging Spectroradiometer (MODIS) considered multiple physiological processes (e.g., photosynthesis, and leaf and root respiration) and the relationship between light use efficiency and environmental factors. Thus, MOD17 improved the accuracy of NPP estimation and was commonly used to analyze the spatiotemporal patterns of NPP on regional and global scales (Running et al., 1999,2004; Turner et al., 2006). However, MOD17 did not address the issue of cloud contamination in the fraction of photosynthetically active radiation (FPAR)/LAI products it used (MOD15A2) (Zhao, Heinsch, Nemani, & Running, 2005; Zhao & Running, 2010). Therefore, in order to promote the sustainable development of ecosystems, we should comprehensively study the coupling response mechanism of NPP to land use and climate changes on the basis of a more accurate estimation of NPP.

Future climate change will lead to a wetter climate in humid regions and a drier climate in arid and semi‐arid regions (MA, 2005). Compared with the humid regions, the eco‐environment in arid and semi‐arid regions is more fragile, with a faster population growth. Environmental change and human interference will further aggravate the ecological imbalance in arid and semi‐arid regions, resulting in more serious or even irreversible negative impacts (Liu et al., 2019; MA, 2005). Thus, it is urgently needed to study the response of NPP to land use and climate changes in these regions. To this end, using the middle‐reaches of the Heihe River Basin (MHRB) in northwestern China as an example, the objectives of this study are as follows: (a) to analyze the spatiotemporal pattern changes of the NPP in the MHRB from 2000 to 2014; (b) to reveal the response mechanism of NPP to land use and climate changes; (c) to discuss the reasons for the spatial heterogeneity of the response mechanism of NPP; and to provide strategies for the sustainable development of ecosystem.

## 2 MATERIALS

### 2.1 Study area

**Figure 1:**
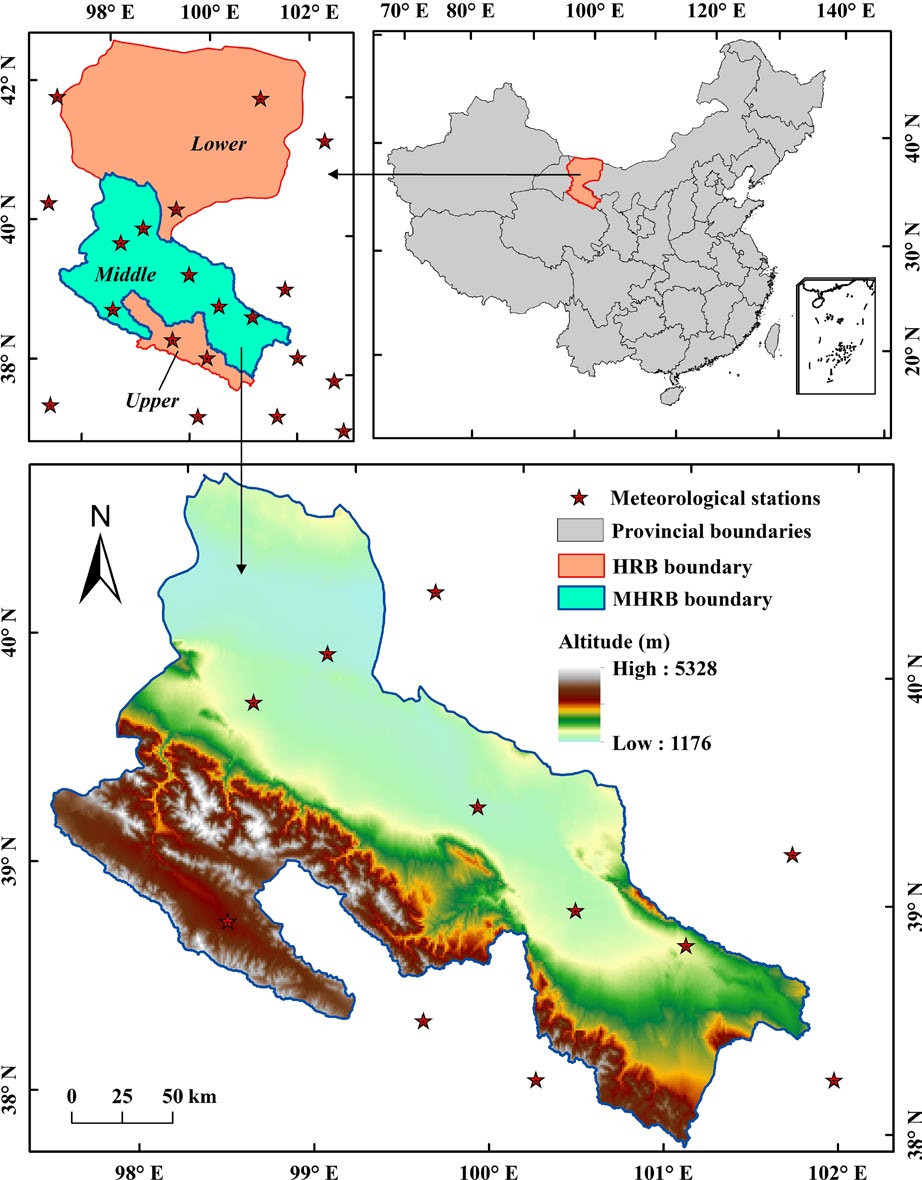
Location of the middle‐reaches of the Heihe River Basin

The Heihe River Basin (HRB) is the second largest inland river basin in the arid and semi‐arid regions of northwestern China, located in the middle of the Silk Road Economic Belt (Figure 1). The upper, middle, and lower reaches of the HRB are water conservation areas, agricultural oasis areas, and ecological conservation areas, respectively. The MHRB is one of the top 10 commodity grain bases in China by virtue of its fertile soil, abundant sunshine, and convenient irrigation. The climate in MHRB is a typical temperate continental climate, with an average annual rainfall of 100–250 mm (70% of the precipitation are concentrated in June–August), an average annual temperature of 6–8°C, an annual sunshine duration of over 3,000 hr, and an annual potential evapotranspiration of 1,600–2,400 mm (Liu, Song, & Deng, 2016; Song, Liu, Deng, Zhang, & Han, 2018). Water is the key factor constraining the development of the ecosystem and human society in the MHRB, in which agriculture consumes more than 90% of the water resources (Liu, Song, & Deng, 2017). Land use and climate changes affect the supply and distribution of water resources, resulting in a change in the ecosystem state of MHRB (Song, Liu, Arowolo, Zhang, & Xu, 2018a; Tan & Zheng, 2017). Changes in temperature and precipitation have enhanced snowmelt, which have led to a slight increase in available water resources of MHRB. However, the increase in agricultural and urban water consumption has led to ecological water reduction and ecosystem degradation (Liu, Song, & Deng, 2017; Song, Liu, Arowolo et al., 2018a).

### 2.2 Data sources

The NPP data used in this study were the MODIS MOD17A3 product, a level 4 version‐55 product at an annual interval, with a spatial resolution of 1,000 m (https://modis.gsfc.nasa.gov/). MOD17A3 developed by NTSG/UMT has effectively eliminated the cloud pollution in the version‐4 NPP product of NASS and has passed the stage‐3 accuracy evaluation, which can be applied systematically and firmly (Running et al., 1999; Zhao et al., 2005). We collected two images of the MHRB each for 2000 and 2014, and their row/column numbers were 25/4 and 25/5, respectively.

The LUC data used in this study were from the Climate Change Initiative Land Cover dataset (CCI_LC) (https://www.esa-landcover-cci.org/), which has an annual interval and a spatial resolution of 300 m. CCI_LC maps were produced by the European Space Agency based on AVHRR HRPT, SPOT‐Vegetation, and PROBA‐V remote sensing images, using the UN Land Cover Classification System as a classification system and containing 22 land cover types (Li, MacBean et al., 2018). We collected the CCI_LC data of MHRB in 2000 and 2014.

The climatic factors used in this study include the annual average temperature and annual precipitation of MHRB in 2000 and 2014, which were the ground observation data from 20 meteorological stations (http://data.cma.cn/) (Figure 1). We interpolated these observations into 1 km raster data based on the thin plate smoothing spline package developed by the Australian National University (ANUSPLIN) (https://fennerschool.anu.edu.au/research/products/anusplin) (Liu, Song, & Deng, 2017).

## 3 METHODS

### 3.1 NTSG MOD17A3 algorithm

Compared with the previous NASA MOD17 algorithm (Running et al., 1999,2004), the NTSG MOD17A3 algorithm has made numerous improvements such as spatial interpolation, cloud pollution removal, and biome parameter recalibration (Zhao et al., 2005; Zhao & Running, 2010). Thus, the version‐55 NPP product developed by NTSG is more accurate and reliable than version‐4 product. The NTSG MOD17A3 algorithm incorporates multisource data into the light use efficiency‐based model to estimate NPP (Zhao et al., 2005; Zhao & Running, 2010).

(1) $$\text{NPP}=\text{GPP}-R_{m}-R_{g},$$

(2) GPP=ε×FPAR×PAR,

where NPP is equal to the gross primary production (GPP) subtracts the maintenance (*R*_m_) and growth respiration costs (*R*_g_). GPP depends on the active radiation use efficiency (*ε*), the FPAR, and the photosynthetically active radiation (PAR). FPAR is from the satellite‐derived MOD15 product, and PAR is from the independent estimates of the Global Modeling and Assimilation Office in NASA (GMAO/NASA).

(3) $$\varepsilon =\varepsilon_{\text{max}}\times {\text{TMIN}}_{s}\times {\text{VPD}}_{s}.$$

As two stress factors, minimum temperature (TMIN_s_) and vapor pressure deficit (VPD_s_) attenuate the maximum radiation conversion efficiency (*ε*_max_) to produce the final *ε*.

(4) $$R_{m}={\text{FR}}_{m}+{\text{LR}}_{m}=\left(F_{w}\times {\text{FRB}}_{m}+L_{w}\times {\text{LRB}}_{m}\right)\times e^{\left[\left(T_{\text{avg}}-20\right)/10\right]},$$

(5) $$R_{g}=0.25\times \text{NPP,}$$

where *R*_m_ consists of the maintenance respiration costs for fine roots (FR_m_) and leaves (LR_m_). *R*_g_ is empirically parameterized as 25% of NPP. *F*_w_ and *L*_w_ refer to the weights of fine roots and leaves, respectively. FRB_m_ and LRB_m_ refer to the maintenance respiration per unit fine root and leaf carbon at 20°C, respectively. *T*_avg_ is the average temperature.

### 3.2 Reclassification of land use

Based on the previous research (Liu et al., 2019), we regrouped the land cover types of the CCI_LC classification system into six land use types, namely, urban land, cropland, forested areas, grassland, water areas, and unused land (Table 1). The reclassification was performed on the User Tool 3.14, a software developed by the European Space Agency that can be used for coordinate transformation, reclassification, and resampling.

**Table 1:** Land use codes and types of reclassification system

Our land use legend	Climate Change Initiative land cover legend
1, Urban land	190, Urban areas
2, Cropland	10, Rainfed cropland; 20, Irrigated cropland; 30, Mosaic cropland
3, Forested areas	40, 100, Mosaic tree and shrub; 50, 60, 70, 80, 90, Tree cover; 110, Mosaic herbaceous cover; 120; Shrubland
4, Grassland	130, Grassland
5, Water areas	160, 170, 180, Flooded vegetation; 210, Water bodies; 220, Permanent snow and ice
6, Unused land	140, Lichens and mosses; 150, Sparse vegetation; 200, Bare areas

Note

The numbers refer to the codes of different land use types, for example, “1” refers to urban land.

### 3.3 Mapping of land use intensity

According to the degree of human activity interference, previous research divided the six main land use types into four grades: Namely, the grading index of unused land was 1, that of forested areas, grassland, or water areas was 2, that of cropland was 3, and that of urban land was 4 (Li et al., 2017). In order to match the spatial resolution of NPP, we measured the land use intensity (LUI) at 1‐km spatial resolution. Each grid contained multiple land use pixels (300 m), and its LUI was calculated based on a weighted summation method. The LUI values range from [100, 400]; the greater its value, the stronger the human interference. To make LUI more indicative, we further divided its values into three grades (namely, 100–200, 200–300, and 300–400 refer to slight, medium, and high LUI, respectively).

(6) $${\text{LUI}}_{x}=100\times \sum_{i=1}^{6}\left(G_{i}\times P_{i,x}\right),$$

where LUI*_x_* denotes the LUI of grid *x*, *G_i_* denotes the grading index of land use *i*, and *P_i,x_* denotes the area proportion of land use *i* on the grid *x*.

## 4 RESULTS

### 4.1 Spatiotemporal pattern changes of NPP

**Figure 2:**
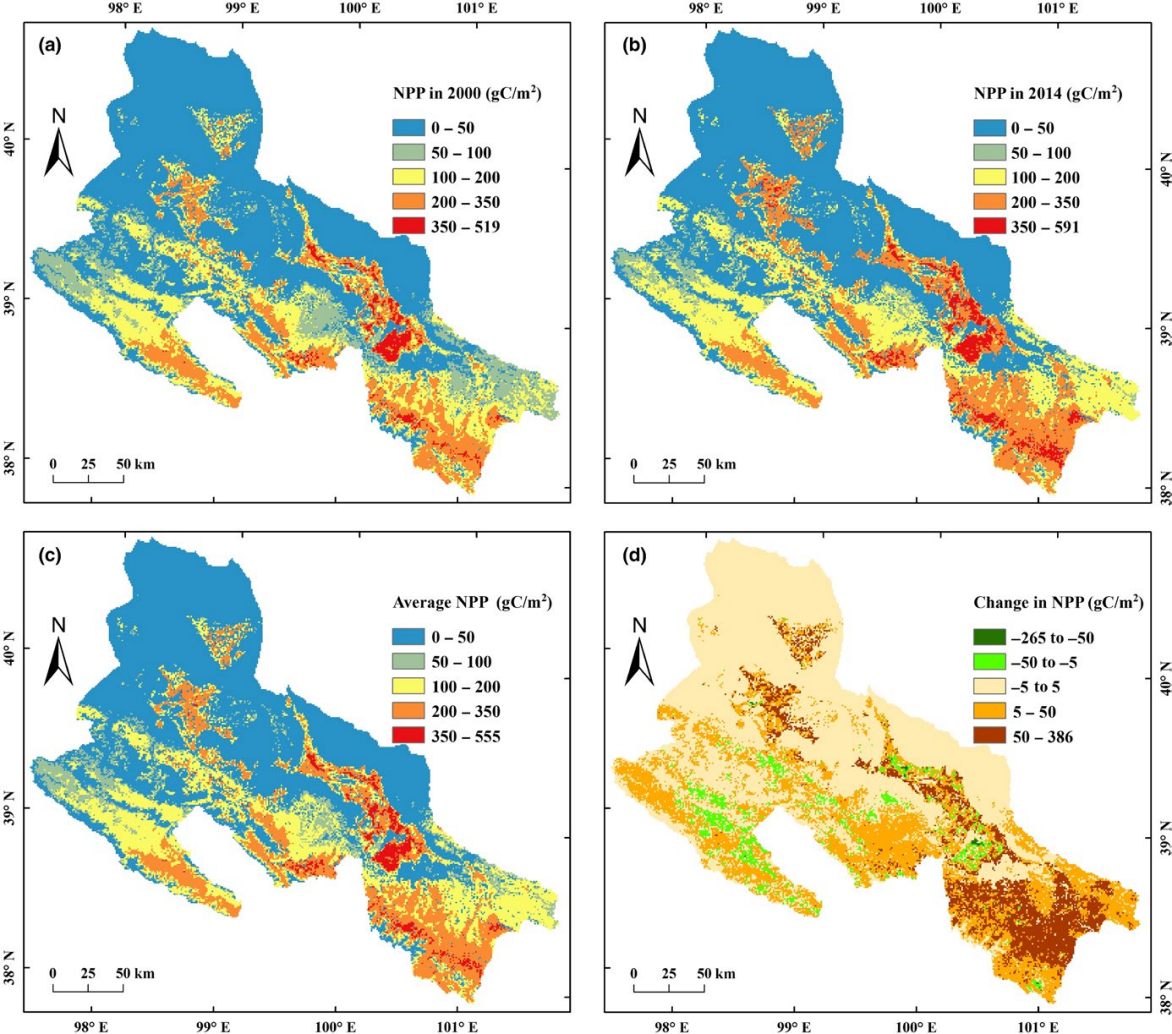
Spatiotemporal patterns of annual net primary production (NPP) in the middle‐reaches of the Heihe River Basin (MHRB): (a) NPP in 2000; (b) NPP in 2014; (c) average NPP from 2000 to 2014; (d) change in NPP from 2000 to 2014

**Figure 3:**
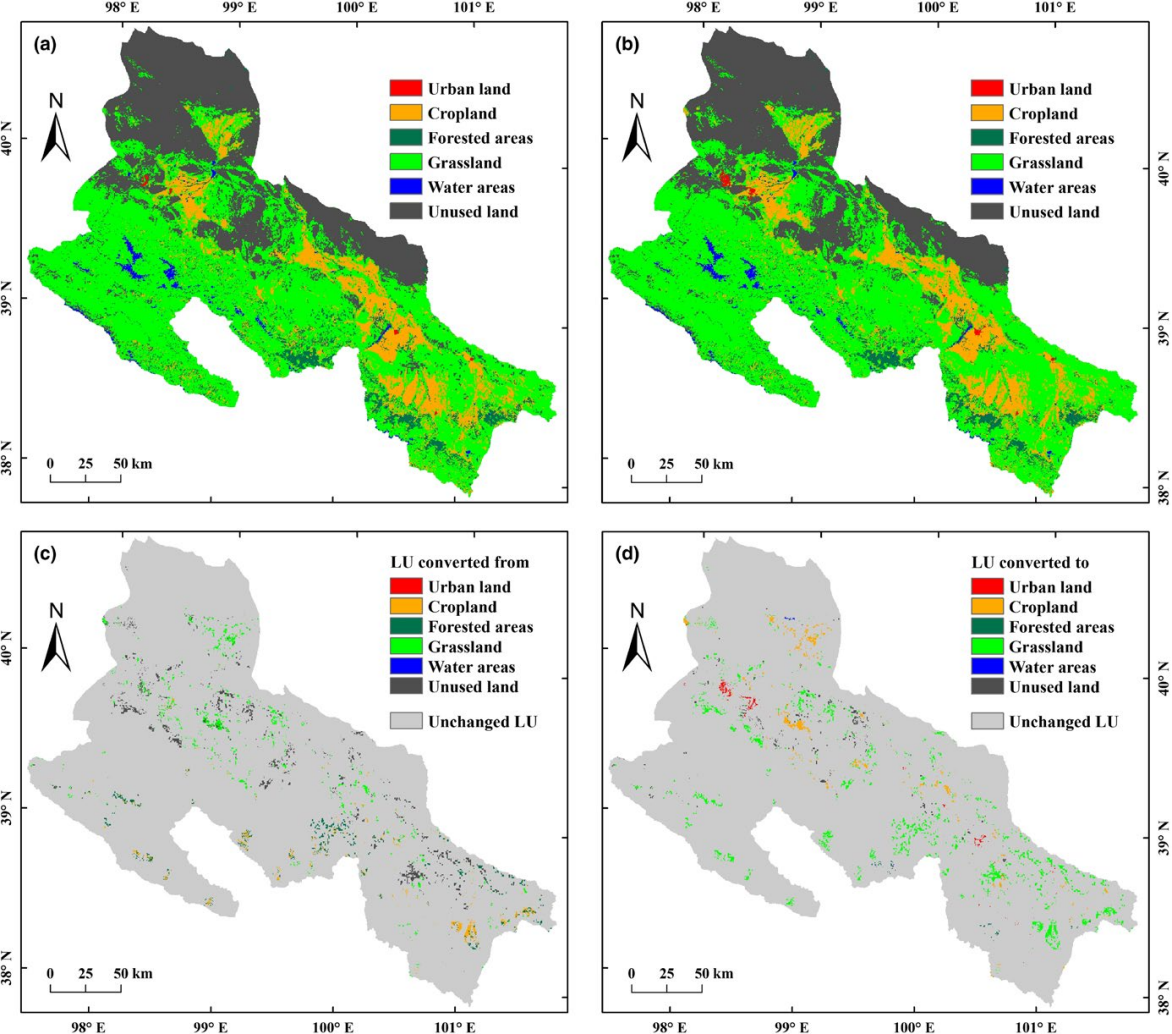
Spatiotemporal patterns of land uses and their transition in the middle‐reaches of the Heihe River Basin (MHRB): (a) land use in 2000; (b) land use in 2014; (c) land use losses from 2000 to 2014; (d) land use gains from 2000 to 2014

From 2000 to 2014, the annual NPP in the MHRB showed a significant spatial heterogeneity (Figure 2). High NPP areas (>350 gC/m^2^) were mainly located in the agricultural oasis of middle low‐altitude plains and in the forested areas of southeastern high‐altitude mountains (Figures 2 and 3). Median NPP areas (100–350 gC/m^2^) were mainly in the high‐altitude cropland and grassland, and the marginal cropland in the central MHRB. Low NPP areas (<100 gC/m^2^) were distributed in the urban land, unused land, and water areas.

Compared to 2000, high NPP areas has expanded in 2014 (Figure 2a,b). The central cropland had higher NPP than the southeastern cropland (Figure 2c). The NPP increase in cropland and forested areas was largest, especially in the southeastern MHRB (Figure 2d). The NPP increase in the central cropland was greater than the marginal cropland (Figure 2d). The high‐altitude grassland had a higher NPP increase than the low‐altitude one. The urban expansion area had a significant reduction in NPP, while some of the central cropland and the high‐altitude grassland had a slight decrease in NPP.

### 4.2 Response of NPP to land use transitions

From 2000 to 2014, the transitions of unused land and sparse grassland into cropland were mainly occurred in the marginal of agricultural oasis (Figure 3), which has led to an increase of 67.4 km^2^ (1.16%) in cropland area (Table 2). The grassland area increased by 597.8 km^2^ (2.33%) due to its gains from unused land and marginal cropland. The urban land area expanded by 79.4 km^2^ (176.84%), which mainly consumed the cropland and unused land around the city. The forested areas in the low‐altitude foothills with lower NPP were shifted to grassland and cropland, which caused an area decrease of 274.6 km^2^ (11.41%). The unused land decreased by 475.4 km^2^ (3.60%) and was mainly transferred to urban land, cropland, and grassland.

**Table 2:** Changes in net primary production (NPP) and land area of different land uses in the middle‐reaches of the Heihe River Basin (MHRB) from 2000 to 2014

Land use types	NPP (gC/m^2^)			Land area (km^2^)		
	2000	2014	Change	2000	2014	Change
Urban land	78.70	72.85	−5.85	44.9	124.3	+79.4
Cropland	203.29	238.76	+35.47	5,727.7	5,794.1	+66.4
Forested areas	185.78	229.53	+43.75	2,406.9	2,132.3	−274.6
Grassland	83.32	97.87	+14.55	25,675.5	26,273.3	+597.8
Water areas	27.28	33.25	+5.97	497	503.4	+6.4
Unused land	2.61	2.25	−0.36	13,197.3	12,721.9	−475.4
MHRB	79.88	94.50	+14.62	47,549.3	47,549.3	0.0

The average NPP values of different land uses were ordered by cropland > forested areas > grassland > urban land > water areas > unused land (Table 2). Compared to 2000, the NPP of forested areas and cropland increased by 23.55% and 17.45%, while that of urban land and unused land decreased by 7.43% and 13.79%, respectively. Under the joint influences of the transitions and NPP changes in different land uses, the average NPP in the MHRB increased by 18.30% (14.62 gC/m^2^).

### 4.3 Response of NPP to LUI changes

**Figure 4:**
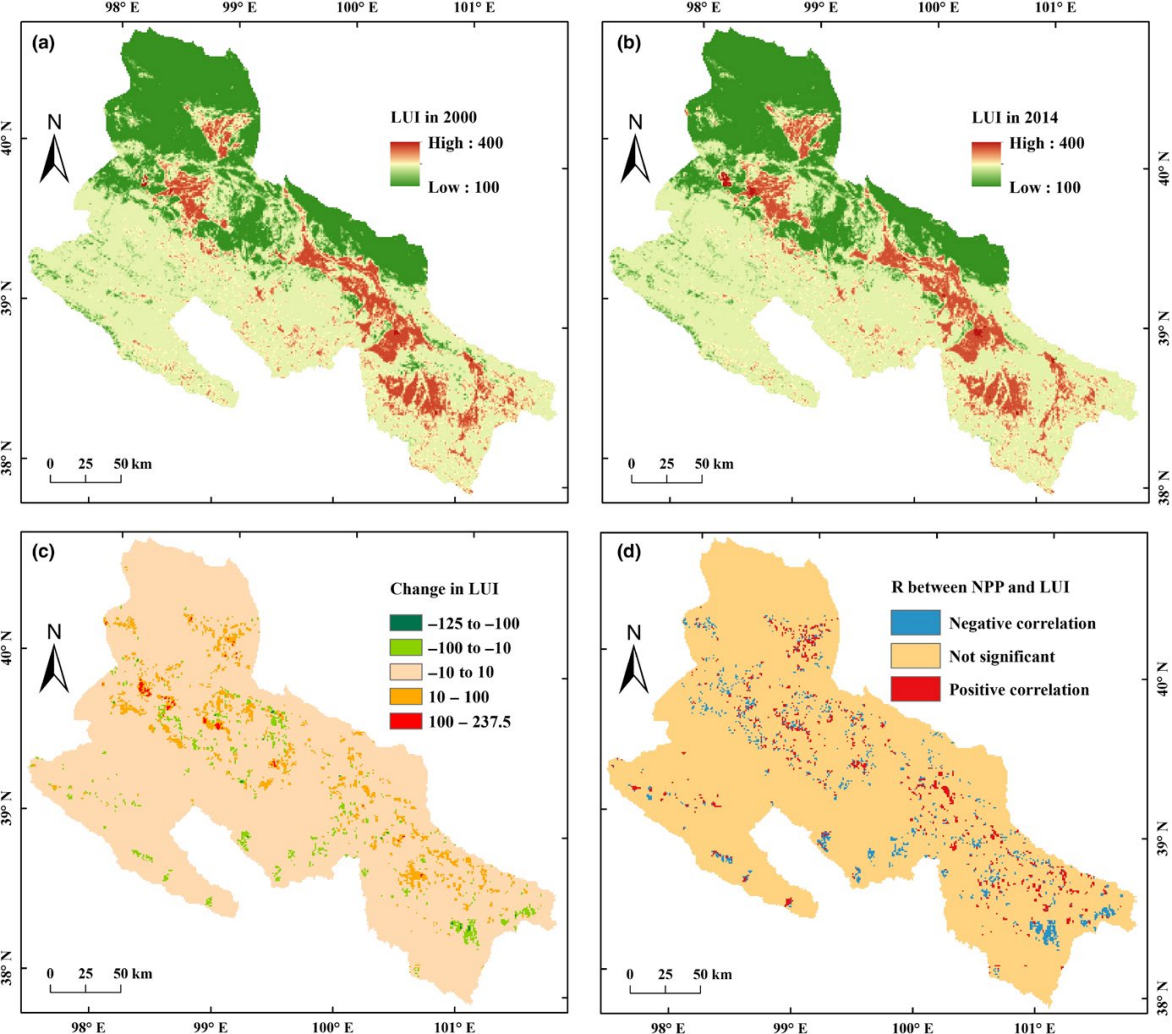
Spatiotemporal patterns of land use intensity (LUI) and its correlation with net primary production (NPP) in the middle‐reaches of the Heihe River Basin (MHRB): (a) LUI in 2000; (b) LUI in 2014; (c) change in LUI from 2000 to 2014; (d) correlation between LUI and NPP

From 2000 to 2014, the land use changes in the MHRB have promoted the average LUI from 184.46 to 185.92 (Table 3). The LUI values of urban land and cropland were relatively large (Figure 4a,b). The expansion of urban land and cropland led to an increased LUI (Figure 4c). The reduced LUI values were mainly occurred in the areas where cropland was returned or abandoned to forested areas, grassland, and unused land (Figure 4c).

**Table 3:** Changes in human and natural factors in the middle‐reaches of the Heihe River Basin from 2000 to 2014

Factors	Unit	2000	2014	Average	Change
Land use intensity	–	184.46	185.92	185.19	1.46
Temperature	°C	5.38	5.96	5.67	0.58
Precipitation	mm	160.92	176.68	168.80	15.76

The responses of NPP to LUI changes presented an obvious spatial heterogeneity (Figure 4d). The conversions of unused land to cropland, forested areas, and grassland, and the conversion of grassland to cropland have led to an increase in both LUI and NPP (positive correlation). LUI and NPP both decreased (positive correlation) in the areas where cropland, forested areas, and grassland were transferred to unused land. Part of low‐yield cropland in the marginal agricultural oasis and mountainous areas was returned or abandoned to forested areas and grassland, resulting in increased NPP but decreased LUI (negatively correlation).

**Figure 5:**
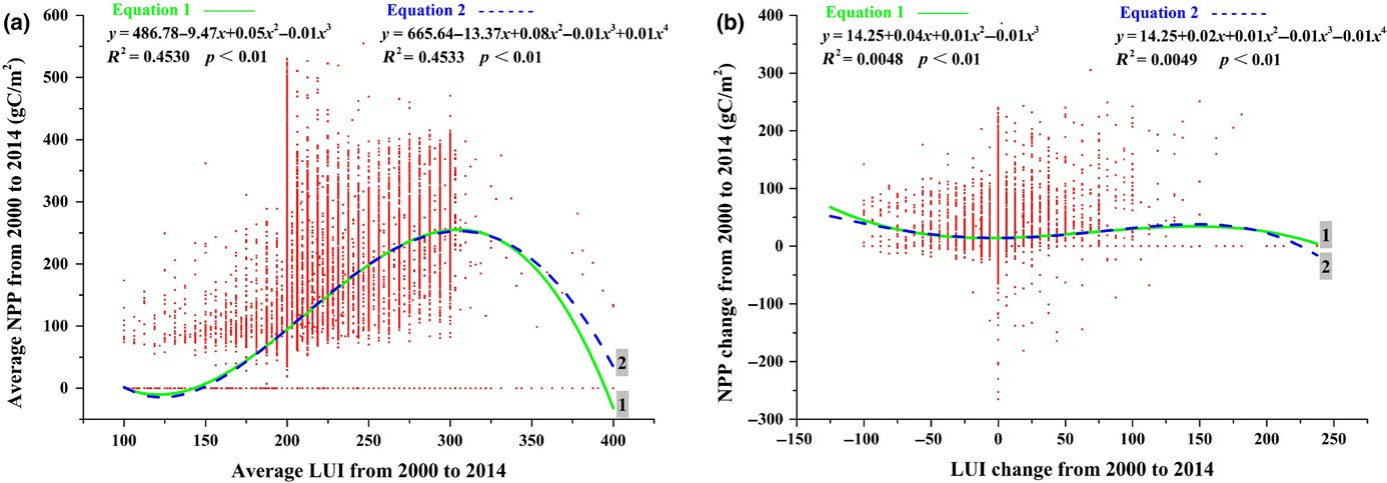
Relationship between net primary production (NPP) and land use intensity (LUI) in the middle‐reaches of the Heihe River Basin (MHRB): (a) average NPP and average LUI; (b) NPP change and LUI change

From 2000 to 2014, the highest and lowest NPP were occurred in the areas with LUI around 200 (forested areas) and around 100 (unused land), respectively (Figure 5a). Along with the increase in LUI, NPP increased when LUI was between 100 and 300, while NPP decreased when LUI was above 300. Generally, the LUI changes (increase or decrease) in the MHRB promoted NPP to a certain extent, and the increase in amplitude of NPP was greater when LUI increases (Figure 5b).

### 4.4 Response of NPP to temperature changes

**Figure 6:**
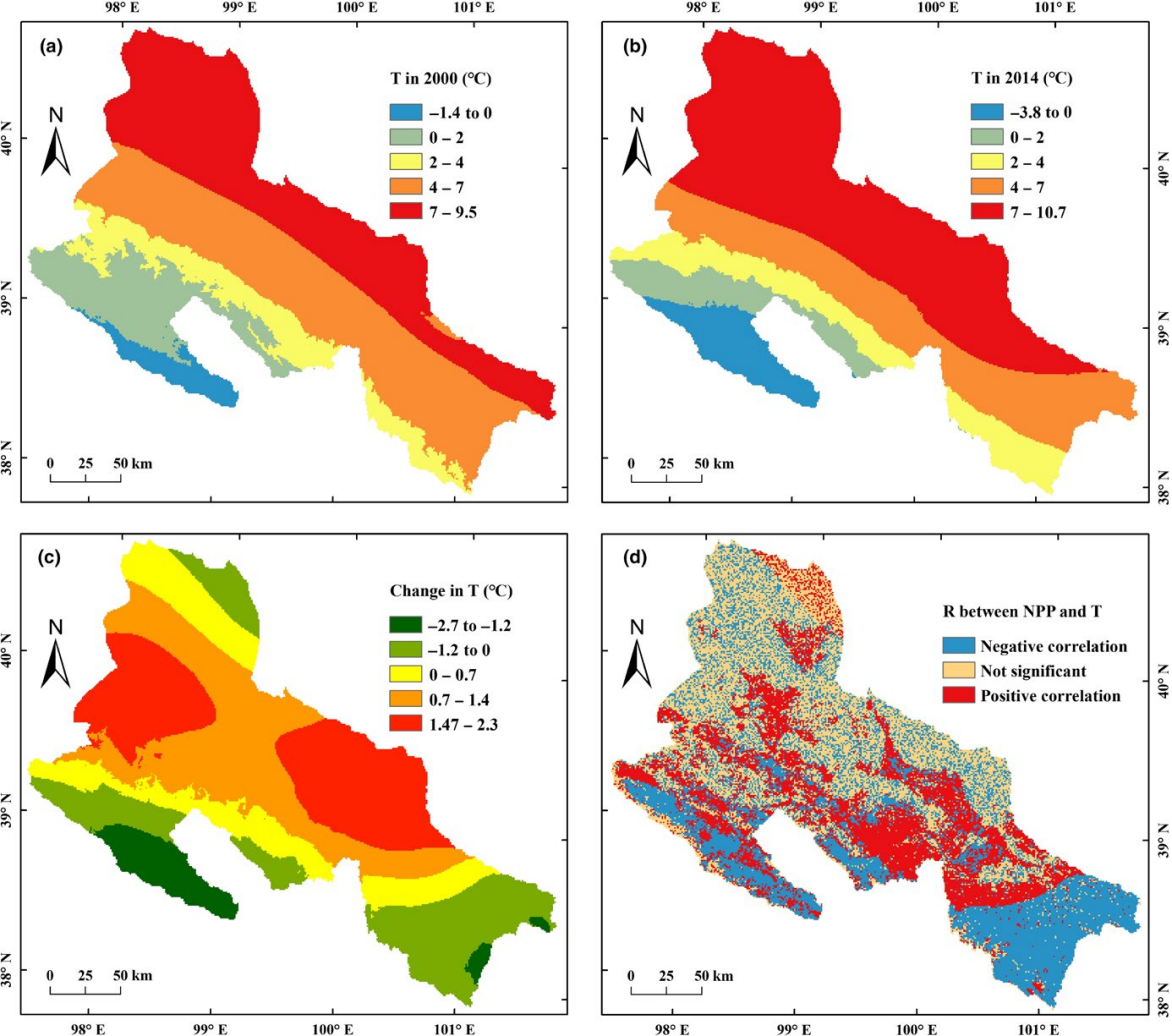
Spatiotemporal patterns of average annual temperature in the middle‐reaches of the Heihe River Basin (MHRB): (a) temperature in 2000; (b) temperature in 2014; (c) change in temperature from 2000 to 2014; (d) correlation between temperature and net primary production (NPP)

The annual average temperature in the MHRB increased from southwest to northeast (Figure 6). From 2000 to 2014, the low‐temperature region (<0°C) has increased slightly, while the high‐temperature region (>7°C) has significantly expanded (Figure 6a,b). Consequently, the average temperature in the MHRB increased to 0.58°C (10.78%) (Table 3). The temperature in high‐elevation area was below 0°C and decreased significantly (Figure 6c). The northeast corner of MHRB also showed a decreasing temperature. The increase in temperature above 0.7°C was mainly occurred in the middle MHRB. The two major urban expansion areas (Zhangye and Jiuquan‐Jiayuguan) had the largest increase in temperature above 1.4°C.

In the temperature increasing areas, the NPP values of most cropland, forested areas, and grassland increased (positive correlation) (Figure 6d). However, temperature increasing led to a decline NPP (negative correlation) in sparse grassland and desert in the extremely arid areas. In the low‐temperature region, the NPP of most forested areas and grassland increased despite decrease in temperature (negative correlation).

**Figure 7:**
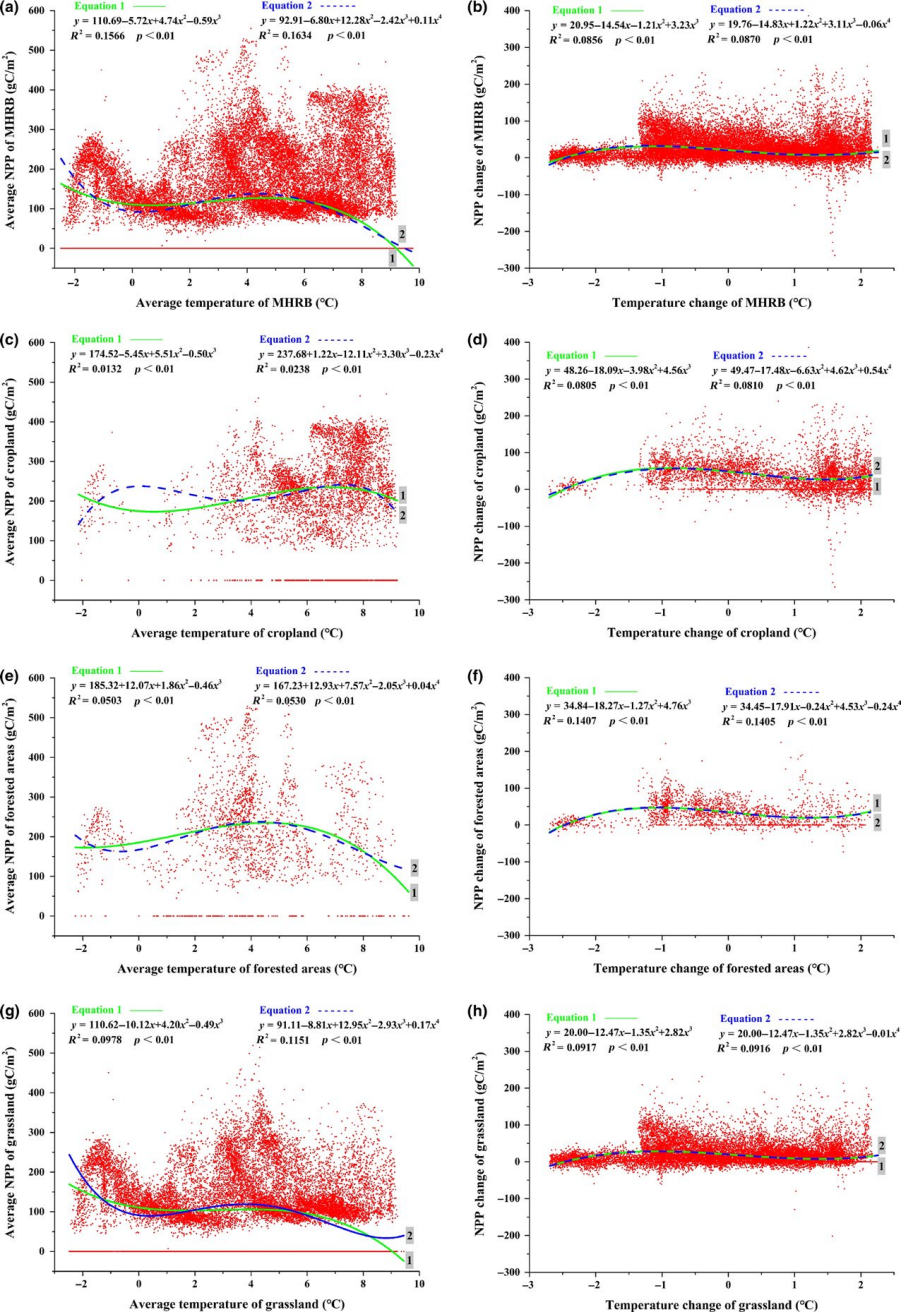
Relationship between net primary production (NPP) and temperature in the middle‐reaches of the Heihe River Basin (MHRB): (a) average NPP and average temperature of MHRB; (b) NPP change and temperature change of MHRB; (c) average NPP and average temperature of cropland; (d) NPP change and temperature change of cropland; (e) average NPP and average temperature of forested areas; (f) NPP change and temperature change of forested areas; (g) average NPP and average temperature of grassland; (h) NPP change and temperature change of grassland

From 2000 to 2014, the high NPP values of MHRB were concentrated at 4–8°C temperature, and the highest NPP was observed at 4°C temperature (Figure 7a). High cropland NPP values appeared at 8°C temperature (Figure 7c), while the high NPP values of forested areas and grassland were occurred at 4°C temperature (Figure 7e,g). When temperature was 0–5°C, the NPP of MHRB increased with increasing temperature. However, when temperature was above 5°C or below 0°C, NPP decreased with increasing temperature. Generally, the NPP of MHRB increased when temperature increased or the decrease in amplitude was <1.2°C (Figure 7b,d,f,h).

### 4.5 Response of NPP to precipitation changes

**Figure 8:**
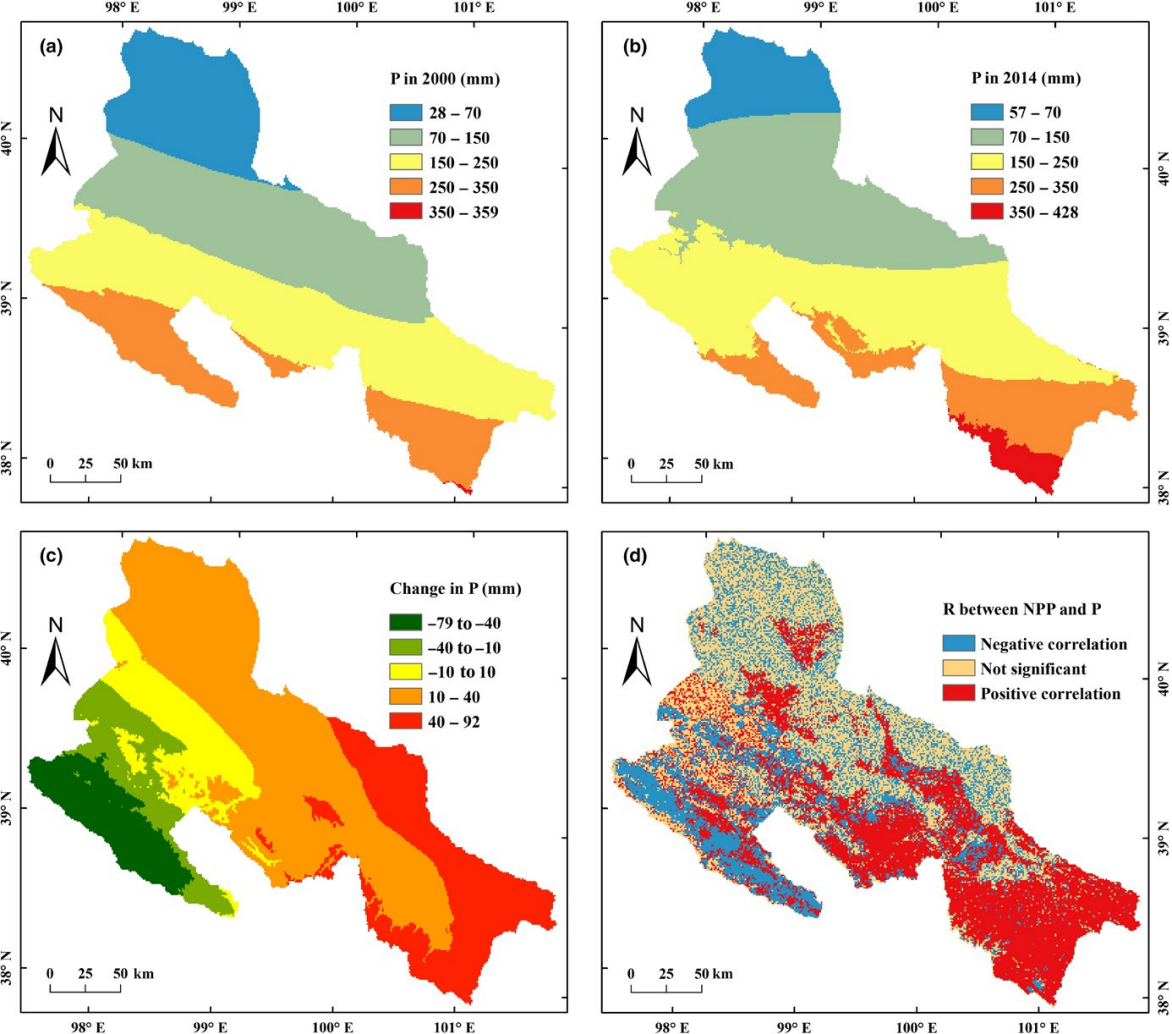
Spatiotemporal patterns of annual precipitation in the middle‐reaches of the Heihe River Basin (MHRB): (a) precipitation in 2000; (b) precipitation in 2014; (c) change in precipitation from 2000 to 2014; (d) correlation between precipitation and net primary production (NPP)

The annual precipitation in the MHRB decreased from southwest to northeast (Figure 8). From 2000 to 2014, the areas with precipitation above 150 mm have expanded (Figure 8a,b). Consequently, the annual precipitation of MHRB increased by 15.76 mm (9.79%) (Table 3). The changes in annual precipitation showed a decreasing trend from southeast to southwest (Figure 8c). The precipitation of MHRB increased in the low‐elevation agricultural oasis, while decreased in the high‐elevation mountainous areas. The largest increase in precipitation (>40 mm) was in the southeast, while the largest decline (<−40 mm) was in the southwest.

In the precipitation increasing areas, the NPP values of most cropland, forested areas, and grassland increased (positive correlation), while that of most unused land (arid desert) decreased slightly (negative correlation) (Figure 8d). In the southwest areas with a relatively high precipitation, although both precipitation and temperature decreased, the NPP of most cropland and forested areas still increased slightly (negative correlation).

**Figure 9:**
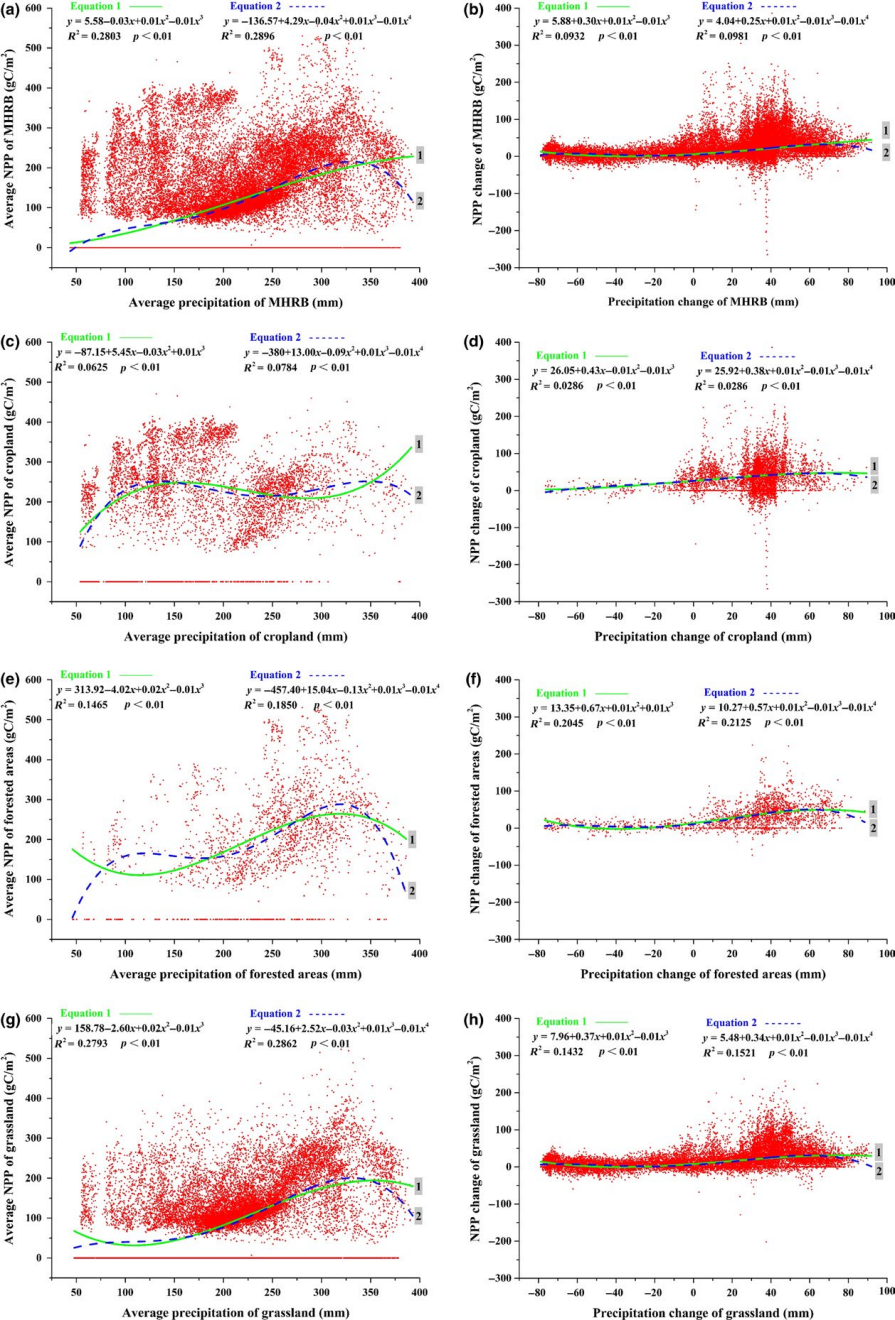
Relationship between net primary production (NPP) and precipitation in the middle‐reaches of the Heihe River Basin (MHRB): (a) average NPP and average precipitation of MHRB; (b) NPP change and precipitation change of MHRB; (c) average NPP and average precipitation of cropland; (d) NPP change and precipitation change of cropland; (e) average NPP and average precipitation of forested areas; (f) NPP change and precipitation change of forested areas; (g) average NPP and average precipitation of grassland; (h) NPP change and precipitation change of grassland

From 2000 to 2014, the highest NPP of MHRB was observed at 300 mm precipitation (Figure 9a). The fluctuation of NPP was higher in the low‐precipitation regions. High cropland NPP values appeared at 0–200 mm precipitation (Figure 9c), while the high NPP values of forested areas and grassland were occurred at 250–350 mm precipitation (Figure 9e,g). When precipitation was 0–350 mm, NPP increased with increasing precipitation. However, when precipitation was above 350 mm, NPP decreased with increasing precipitation due to the accompanying significant temperature decrease. Generally, the NPP in the MHRB increased with increasing precipitation (Figure 9b,d,f,h). The increase in amplitude of NPP reduced when the increase in amplitude of precipitation increases. The increase in NPP was highest at a precipitation increase of 40 mm.

## 5 DISCUSSION

### 5.1 Spatial heterogeneity of NPP response to land use and climate changes

Land use and climate changes jointly determined the spatiotemporal pattern of NPP and its changes. Driven by economic benefits, farmers in the MHRB reclaimed more cropland and adjusted the crop planting structure (Liu et al., 2016). With the help of artificial irrigation and other agricultural inputs, the NPP of cropland was generally larger than that of forested areas and grassland in the arid and semi‐arid regions. Newly expanded cropland was mainly converted from unused land and sparse grassland, resulting in increased LUI and NPP in the marge of agricultural oasis. Moreover, the planting adjustment of wheat and barley (C3 crop with relatively low NPP) into corn (C4 crop with relatively high NPP) led to a NPP increase in the central agricultural oasis. Due to the higher accumulated temperature and more convenient irrigation in the central MHRB (Liu, Song, & Deng, 2017), this area had a higher increase in cropland NPP than high‐altitude mountainous. Meanwhile, influenced by the Grain‐for‐Green project, farmers tended to returned low‐yield cropland to forested areas and grassland, resulting in decreased LUI but increased NPP in the marginal agricultural oasis and mountainous areas of MHRB. On the contrary, affected by economic and population growth, the urban sprawl of MHRB caused a reduction of cropland, forested areas, and grassland around cities (Liu et al., 2019) and an increase in LUI. Consequently, the NPP of newly expanded urban land decreased (Li, Wang et al., 2018). However, due to the expansion of urban green space, the NPP in urban centers increased (Yan et al., 2018).

Previous research confirmed that climate change plays the major role in NPP variations in the arid and semi‐arid regions (Lai et al., 2018; Li, Wang et al., 2018). Generally, the climate warming and wetting in the MHRB were conducive to NPP increase. A certain degree of warming facilitated the photosynthesis of thermophilic corn in the central MHRB (Liu et al., 2016) and promoted its NPP. Moreover, warming boosted the snowmelt of MHRB (Song, Liu, Deng et al., 2018), which was beneficial to NPP increase. Meanwhile, the temperature decreasing <1.2°C also promoted the NPP of hardy forest, grass, and crops (wheat, barley, and rapeseed) in high‐altitude mountains, due to the simultaneous precipitation increase. In addition, rural–urban migration has weakened the human activities (e.g., wood cutting and grazing) in mountain areas (Li et al., 2017; Li & Tan, 2018; Xiao, Hu, Tan, Li, & Li, 2018), which further increased the NPP in forested areas and grassland. However, warming‐induced water stress compromised photosynthesis (Zhao & Running, 2010), leading to a decline NPP in arid sparse grassland and desert areas. The decrease in both temperature and precipitation generally reduced NPP, but the NPP in the well‐protection areas still increased slightly due to ecological conservation measures such as grazing prohibition, livestock reduction, and the Grain‐for‐Green project.

### 5.2 Policy implications for improving ecosystem NPP and sustainability

Although previous studies have confirmed the NPP increase in arid and semi‐arid regions due to the greening of cities (Li, Wang et al., 2018; Yan et al., 2018), urban sprawl‐induced cropland and ecological land losses, and other negative impacts should pay more attention (Liu et al., 2019). Therefore, it is crucial to control urban sprawl and foster urban green space (Liu et al., 2019) for the improvement of NPP and the sustainable development of socio‐ecological systems. Besides, cropland expansion, which was mainly from unused land and grassland, improved the NPP of MHRB. However, most of the cropland NPP was appropriated by human society and consumed huge water. Thus, cropland expansion is an unsustainable way to food security. We should cultivate water‐saving crops (Liu et al., 2016), import high‐virtual‐water agricultural products from water‐rich areas (Liu et al., 2019), improve crop water use efficiency (Song, Liu, Deng et al., 2018), and close crop yield gaps (Lu & Fan, 2013; Xin, Li, Zhu, & Tan, 2009) if we are to achieve food‐water‐ecological security in arid and semi‐arid areas. Furthermore, the conversion of low‐yield cropland to forested areas and grassland increased the NPP of the MHRB. However, previous research has demonstrated that it is worth nothing to widely plant trees and grass in the arid and semi‐arid climatic conditions, because such man‐made NPP growth is generally temporary with limited water resources (Lai et al., 2018; Yin, Pflugmacher, Li, Li, & Hostert, 2018). Thus, a comprehensive understanding of NPP response to land use and climate changes is essential to ensure the sustainability of ecological conservation projects.

## 6 CONCLUSION

In this study, we analyzed the spatiotemporal changes of NPP, land use, and climatic factors in the MHRB from 2000 to 2014 and revealed the spatial heterogeneity of NPP responses to land use and climatic changes. The findings of this study provide scientific basis for NPP improvement and sustainable ecosystem management in arid and semi‐arid areas.

Our results confirmed that LUI increased, and climate warming and wetting promoted the NPP in the MHRB. The widespread conversions of unused land and grassland to cropland increased both LUI and NPP. Although cropland expansion boosted NPP, it was not conducive to sustainable ecosystem development due to huge water consumption and human‐appropriated NPP. Urban sprawl, which occupied cropland, forest, and grassland and reduced NPP, should be limited. On the contrary, the conversion of low‐yield cropland to forest and grassland increased NPP, and should be encouraged. In addition, climate change plays the major role in NPP variations, and water availability is the major constraint on NPP. The increase in temperature and precipitation generally improved NPP. The temperature decreasing <1.2°C also promoted the NPP of hardy vegetation due to the simultaneous precipitation increase. However, warming‐induced water stress compromised the NPP in the extremely arid areas. Although cropland experienced worse climate change, its NPP increase was still greater than natural vegetation due to the irrigation, fertilizers, and other artificial inputs it received. The decrease in both temperature and precipitation generally reduced NPP, but the NPP in the well‐protection or less‐disturbance areas still increased slightly.

In order to well adapt the future climatic changes, effective ecosystem management should focus on alleviating or avoiding negative human activities (e.g., excessive expansion of cropland and urban land, deforestation) and strengthening positive ones (e.g., grazing prohibition, reforestation). A comprehensive understanding of the NPP response mechanism to land use and climatic changes provides decision‐makers with the foundation for improving ecosystem NPP and sustainability. However, the interactions between land use and climate changes complicated this response mechanism. How to eliminate the interrelated influences of the two factors and attribute the coupling response mechanism is the focus and difficulty of future ecosystem management.

## CONFLICT OF INTEREST

None declared.

## AUTHOR CONTRIBUTIONS

Minghong Tan and Xingyuan Xiao designed the research. Xingyuan Xiao performed the research and wrote the manuscript. Xiubin Li, Tao Jiang, and Minghong Tan supervised the research and contributed with valuable discussions and scientific advice. Minyue Hu, Yaqun Liu, and Wen Zeng supported the data collection, processing, and analysis. All authors contributed to the discussion and the writing.

## Data Availability

NPP, land use, land use intensity, and meteorological ground observation and interpolation data of the MHRB in 2000 and 2014: Dryad Data Repository (https://doi.org/10.5061/dryad.7238hd3).
